# A Mobile Asset Tracking System Architecture under Mobile-Stationary Co-Existing WSNs

**DOI:** 10.3390/s121217446

**Published:** 2012-12-14

**Authors:** Tae Hyon Kim, Hyeong Gon Jo, Jae Shin Lee, Soon Ju Kang

**Affiliations:** 1 College of IT, Kyungpook National University, 1370 Sankyuk-dong, Buk-gu, Daegu 702-701, Korea; E-Mails: namestrike@ee.knu.ac.kr (T.H.K.); tsana@ee.knu.ac.kr (H.G.J.); 2 ARTSYSTEM, IT Convergence Industry Build., 1370 Sankyuk-dong, Buk-gu, Daegu 702-701, Korea; E-Mail: faithlee3@hanmail.net

**Keywords:** pervasive computing, sensor network architecture, mobile asset tracking

## Abstract

The tracking of multiple wireless mobile nodes is not easy with current legacy WSN technologies, due to their inherent technical complexity, especially when heavy traffic and frequent movement of mobile nodes are encountered. To enable mobile asset tracking under these legacy WSN systems, it is necessary to design a specific system architecture that can manage numerous mobile nodes attached to mobile assets. In this paper, we present a practical system architecture including a communication protocol, a three-tier network, and server-side middleware for mobile asset tracking in legacy WSNs consisting of mobile-stationary co-existing infrastructures, and we prove the functionality of this architecture through careful evaluation in a test bed. Evaluation was carried out in a microwave anechoic chamber as well as on a straight road near our office. We evaluated communication mobility performance between mobile and stationary nodes, location-awareness performance, system stability under numerous mobile node conditions, and the successful packet transfer rate according to the speed of the mobile nodes. The results indicate that the proposed architecture is sufficiently robust for application in realistic mobile asset tracking services that require a large number of mobile nodes.

## 1. Introduction

Recently, the rapid advancement of technology related to wireless sensor networks (WSNs) has increased the development of location-based service fields, such as mobile asset tracking, home networking, environmental control and monitoring, safety systems, and personal health care [1–4].

For mobile asset tracking systems in particular, additional design considerations are required along with the basic WSN system design considerations such as low power consumption and tiny size. First of all, many previous reports have attempted to increase the accuracy of mobile node localization. However, due to the burden of the accuracy factor, numerous mobile nodes could not be supported simultaneously. Since the number of communication nodes can easily amount to thousands in a given service, an effective system architecture is necessary despite limited available resources (*i.e.*, low power, low communication bandwidth). Second, simple but effective methods for supporting the mobility and the location-awareness of communication nodes are critical, although this can be quite difficult to achieve. Especially, under legacy WSN technologies such as ZigBee [5], the entire network can easily break down during heavy traffic and frequent movement of mobile node conditions. Third, it is desirable to reuse the existing WSN network infrastructure in order to minimize installation costs and maintain legacy WSN services. The new architecture should support both legacy WSN services as well as the new mobile asset management service with minimum modifications. Using only the legacy WSN technique, it is not easy to cope with all of the above mentioned requirements. Many previous studies [1–4] have introduced new architectures such as mobility support and precise tracking for mobile asset tracking service. However, in a practical environment, they are insufficient as it is too complicated to support a massive number of mobile nodes. On the contrary, our research focuses on increasing the number of mobile nodes tracked simultaneously using the legacy WSN infrastructure.

Accordingly, we suggest a practical system architecture comprising a communication protocol, a three-tier network, and middleware. The proposed network architecture is divided into three tiers: a mobility tier, a sensor network tier, and a backbone network tier. In the mobility tier, bi-directional location-awareness and asynchronous message delivery protocols (LIDx and LAMD) are placed between the stationary and mobile nodes. The mobile nodes transfer their current location, service-related requests, and response data, such as environmental sensing values and control requests, by sending messages asynchronously to the stationary node that is in the best communicative position. According to the current location of the mobile node, the stationary node can provide various mobile asset tracking services to the mobile nodes located around the stationary node. By enabling bi-directional communication, unlike that found in RFID [6,7], improved and efficient mobile asset tracking services can be attained. In the sensor network tier, we adopted the ZigBee protocol for communication amongst stationary nodes since it provides a stable network implementation for stationary nodes and has been well established in numerous legacy WSN architectures. To provide compatibility between the mobility tier and the sensor network tier, mobile nodes use the same physical and MAC layer as stationary nodes. On the other hand, supporting the mobility of mobile nodes under the ZigBee protocol is a demanding, complex process that can easily disrupt the entire network. Therefore, the proposed architecture attempts to guarantee the mobility of mobile nodes while also minimizing their impact on the pre-established network. In the backbone network tier, the coordinator gateways convert WSN messages received from the stationary nodes to TCP/IP messages for the server side middleware and *vice versa*. The middleware manages the current locations of the mobile nodes in real-time and routes the messages received from a source mobile node to a destination mobile node for an asynchronous message delivery. The reported data, such as current location and environmental sensor values, are saved in an internal database located on the server. Smart devices such as smart phones and smart tablet, can access the service through the server and identify the current location of mobile nodes and then transmit service-specific messages such as “Blink the LED” and “Turn on the buzzer”. This can be done, of course, regardless of the current locations of the mobile nodes and smart devices.

The remaining of this paper is organized in the following manner: Section 2 examines related studies. Section 3 describes the overall system architecture for the tracking service and discusses the detailed design, including the protocol, the service network, and the middleware. Section 4 evaluates the system in a realistic test bed. Finally, Section 5 concludes by summarizing our current work and presenting future directions.

## 2. Related Works

Many researchers [8–11] have attempted to increase mobile node location-awareness by using various types of sensors, such as GPS, RFID and WSN. GPS sensors work well in outdoor environments; however, they suffer from a large number of errors in indoor environments. In addition, GPS sensors consume a great deal of power, making them unsuitable for small mobile assets and battery powered devices. RFID systems are the cheapest solution for stably supporting numerous mobile nodes, but as bidirectional communication is not supported, they cannot provide the desired interactive features in mobile asset tracking services. The RFID reader is a large and expensive device compared to RFID assets, which makes it difficult to use as a mobile device. The WSN system is a good solution for mobile node localization since it consumes a low amount of power and can achieve localization without additional sensors. However, most location-aware techniques [12–14] are focused on the precision of the location with the axis’s of coordination, including the excessive demand of the stationary reference nodes despite too much traffic. Many of these problems are caused by instability of the current WSN technology, including the need for frequent network reconfigurations for updating the *ad-hoc* routing table, due to the free-mobility of the mobile nodes [4]. As mentioned in the Introduction, they are insufficient as it is too complicated. Therefore, a simple protocol is needed as legacy WSN systems are characterized by low computing power and low bandwidth.

Research is currently being conducted in WSN test beds for various applications. There are many test beds for this, including Kansei [15], Sensenet [16], and Mobile Emulab [17]. Each test bed has unique characteristics based on its main purpose. Our test bed is a new implementation, and our design aims to support efficient and stable large-scale mobile asset tracking under mobile-stationary co-existing WSN infrastructures.

## 3. Proposed Systems Architecture for Mobile Asset Tracking Service

### 3.1. Overall System

**Figure 1.**
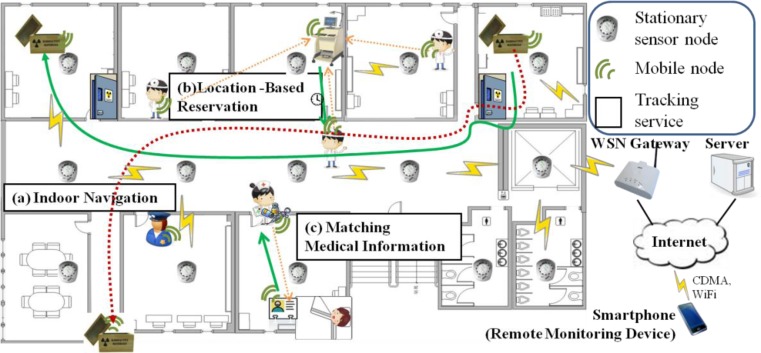
Mobile asset tracking services in a hospital.

Figure 1 describes the mobile asset tracking services that are expected to be provided in a hospital. For instance, in Figure 1(a), the drug box is tracked in real-time on its way to its destination. If the box is found to be in an illegal location, an alarm message is dispatched to the guards nearest the box. In Figure 1(b), a frequently used medical device employs a reservation service in order to make its usage rate more efficient. According to the reservation queue, the device sends a notification message asynchronously to the next user when it becomes available. In Figure 1(c), a matching service between a prescription drug and a patient is employed. Medical staff can play a role in preventing prescription drug misuse and abuse by using this service to confirm whether or not a particular medication is for a particular patient. These mobile assets, *i.e.*, the drug boxes, medical devices, and patients, can move around the environment freely.

It is assumed that a legacy wireless sensor network, including infrastructures such as stationary nodes, has already been deployed in this environment for environmental sensing and control purposes. In order to support the above scenarios, the overall environment is split into multiple unit spaces, such as a room or a floor. Each stationary node is in charge of a unit space in order to monitor its immediate locale. In addition, the stationary node acts as an access point for communication with the mobile nodes (a mobile node and a mobile asset have the same meaning in this paper and are used interchangeably) located in the unit space and is used for the reference location of each mobile node currently in its domain. As mentioned above, mobile nodes can be attached to physical mobile objects, usually in the form of small tags. The mobile asset tracking service entails all communication between the mobile and stationary nodes as well as between the stationary nodes and a server through gateways. The services can be accessed by smart devices, such as smart phones and smart tablet, by using specially designed and optimized service apps.

**Figure 2.**
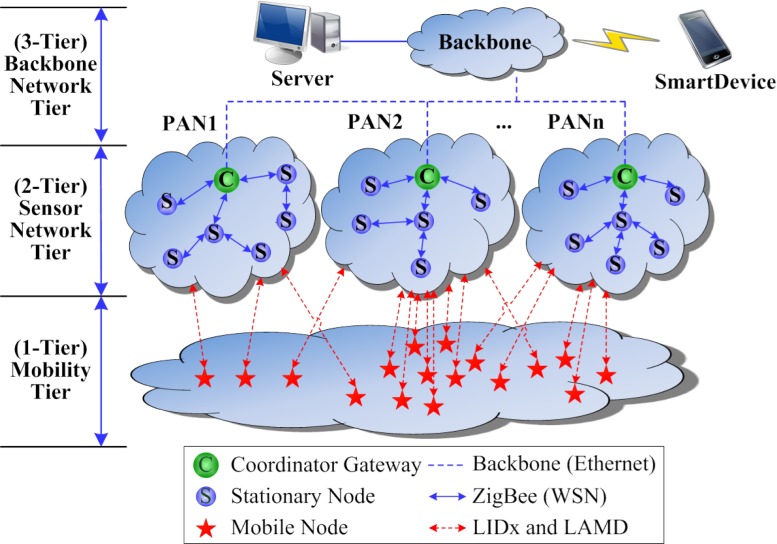
Proposed mobile-stationary co-existing WSN architecture.

Figure 2 shows the proposed mobile-stationary co-existing WSN architecture, which is made up of the mobility tier, the sensor network tier, and the backbone network tier. The nodes in the mobility tier and the sensor network tier commonly use the IEEE 802.15.4 MAC protocol in order to support compatibility with legacy WSN protocols, whereas for the free-mobility of the mobile nodes, the LIDx and LAMD protocols [18–20] are implemented on top of the IEEE 802.15.4 MAC protocol. Mobile nodes do not have a network protocol stack due to memory and power constraints, whereas stationary nodes do possess a network protocol stack. The sensor network tier and the mobility tier are loosely-coupled using the LIDx and LAMD methods, an essential step that assures the free-mobility and free-message-delivery of the mobile nodes. We limit the communication between stationary and mobile nodes to a single hop in order to minimize network congestion and delay problems. In the proposed architecture, movement of the mobile nodes does not impact the sensor network configuration since the mobile nodes do not use the network protocol; they only influence the condition of the physical network when occupying a communication channel. The backbone network tier is connected between the coordinator gateways (CG) and the oserver. The CGs forward the environmental sensing data as well as the location data of the multiple mobile nodes received from the stationary nodes to the server. The CGs also convert the sensor network messages to TCP/IP messages for the server as well as TCP/IP messages to sensor network messages for the stationary nodes. As mentioned above, smart devices are used to maintain the services.

### 3.2. LIDx and LAMD: the Communication Protocol between the Stationary and Mobile Nodes

**Figure 3.**
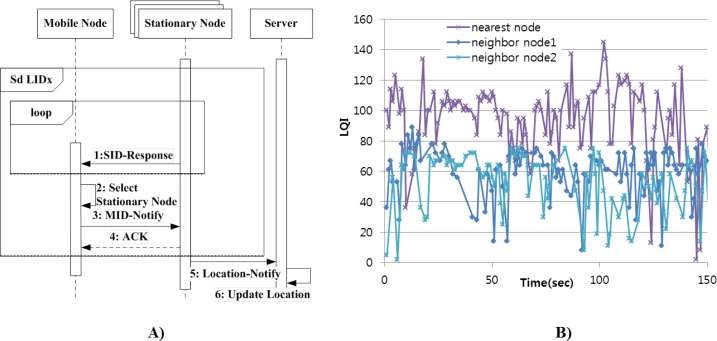
(**A**) Sequence diagram of LIDx protocol and location-updating sequence of mobile node. (**B**) LQI graph between mobile and stationary nodes.

The Location-ID eXchange (LIDx) concept was developed in our previous research [18–20]. The mobility tier supports the LIDx protocol between stationary and mobile nodes in order to achieve real-time location-awareness. Using the LIDx protocol, the mobile nodes can change their location freely. Figure 3(A) is a sequence diagram showing the proposed LIDx protocol between the mobile and stationary nodes. Each mobile node selects its nearest stationary node by comparing the signal strength of packet. Then, it sends its ID to nearby stationary nodes in order to report its current location, after which it sends its ID to the server.

The server then updates the information regarding the location of each mobile node. In the LIDx protocol, the overall environment is split into multiple unit spaces, which are divided physically into rooms or floors. Each stationary node is in charge of a unit space and periodically sends a LIDx packet (a beacon packet). The mobile nodes collect these beacon packets and select the nearest stationary node’s ID based on signal strength of the packets. Figure 3(B) shows the Ling Quality Indicator (LQI) of packets from each stationary node as a mobile node moves into one unit space. As the figure shows, signal strength from the stationary nodes fluctuates due to the interference caused by various obstacles. By definition of unit space, however, neighboring stationary nodes are divided by large obstacle such as wall or dispersed by far distances. Hence, the LQI of packets received from the nearest node is higher compared to those of others. In conclusion, although signal strength is interfered by various types of noise, we can select a nearest stationary node by comparison of signal strength.

**Figure 4.**
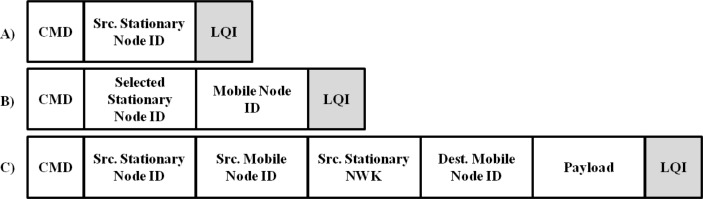
LIDx and LAMD packet formats (partial).

LAMD is an acronym for “LIDx-based Asynchronous Message Delivery”, which is used to exchange asynchronous messages (AMs) amongst mobile nodes through stationary nodes. The protocol can be used to set up active and sleep periods for individual radios in order to obtain longer operating times, as it is critical in WSNs to design devices that consume low amounts of power. Effective use of low data bandwidth is as important as the low power feature. In general, sensor networks support a relatively low data rate (e.g., under 250 Kbps in ZigBee) compared to other communication systems such as Bluetooth or WLAN. Employing mobile nodes in a sensor network may cause a large amount of traffic and insufficient memory, especially in situations where multiple mobile nodes try to access a single stationary node. The proposed protocol is simple enough to be used in the WSN environment. The stationary node broadcasts LIDx packets, and the packet format can be seen in Figure 4(A). The only information required in this packet is the source stationary node’s ID. Each stationary node is assumed to be installed at a known position, which means that the unique ID of each stationary node corresponds to its location information. The LQI values, shaded in Figure 4, are automatically updated by the receiver (either a stationary or mobile node). This value is the main ingredient used to determine the current locations of the mobile nodes. All mobile nodes receiving LIDx packets report their IDs to the nearest stationary node. The packet structure is shown in Figure 4(B). Based on the location information from the mobile nodes, the stationary node appends this additional information and sends it to the server using the legacy sensor network protocol. Mobile nodes also exchange additional information, including source mobile node ID (Src. Mobile Node ID), source stationary node network ID (Src. Stationary NWK), destination mobile node ID (Dest. Mobile Node ID), and payload in the LAMD protocol. This is shown in Figure 4(C).

**Figure 5.**
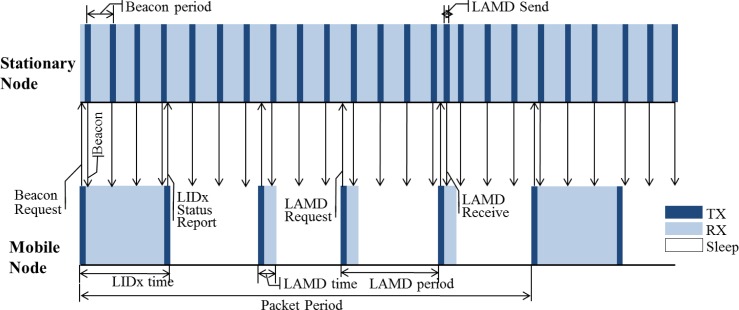
LIDx and LAMD timeline.

Figure 5 shows a timeline between the mobile and stationary nodes operating with the LIDx and LAMD protocols. It shows that the stationary node always has its radio on in order to minimize message transmission delays, whereas the mobile node turns its radio on/off periodically to minimize its power consumption. This is possible since stationary nodes can incorporate hard-wired power and/or large batteries; however, this is not an option for mobile nodes. As shown in Figure 5, each stationary node periodically sends a LIDx packet (a beacon packet). The mobile nodes collect these beacon packets and select the nearest stationary node’s ID based on LQI values of the packets. The mobile node then sends a LIDx status-report packet to the stationary node which is then updated to the server. In regards to the LAMD messages, the mobile node sends a LAMD Request-packet to the stationary nodes through unicast or broadcast methods according to predefined policy. If the stationary nodes have LAMD messages, they send them to the mobile nodes.

### 3.3. The Middleware for Proposed Architecture

In our architecture, all the location information of mobile nodes is centralized in the server. However, services using this information are not only Mobile Asset Tracking/Monitoring; they include Location-based Mobile Resource Reservation, and Message Delivery between mobile nodes as well, especially as remote services. Therefore, in order to provide location information anywhere, we implemented the middleware to the server for common functionalities such as managing location information of the mobile and stationary nodes as well as transferring AMs among the mobile nodes.

**Figure 6.**
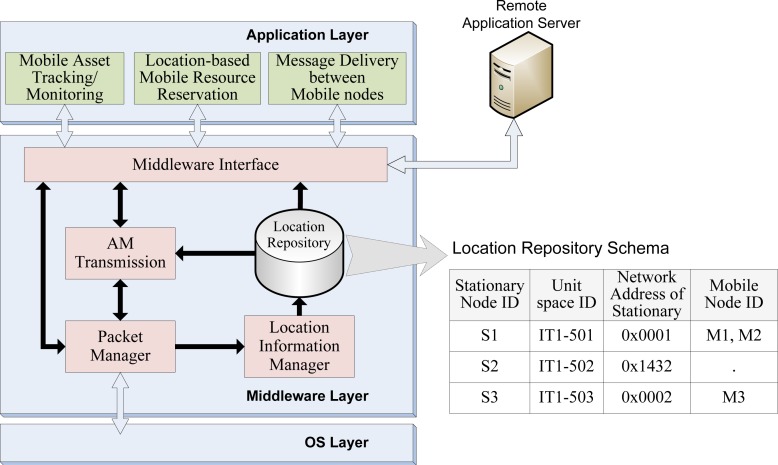
Middleware architecture in the Server.

Figure 6 shows the middleware architecture in the server. The middleware is composed of packet manager, location information manager, AM transmission, and middleware interface. The packet manager routes the packet to each component respectively. The Location Information Manager maintains the Location Repository for location-awareness. The AM transmission determines the target stationary node that most recently detected the destination mobile node using the Location Repository. Detailed description of this operation in this component will be followed. The middleware interface abstracts and provides the functionalities of middleware to services.

When a LAMD message is routed to the server from the source mobile node, the middleware determines the target stationary node that most recently detected the destination mobile node. This means that, all necessary information regarding every stationary node must be specified in the middleware so that the mobile node’s location can be matched to the proposed target location. As Figure 6 shows, the middleware has to maintain four types of information to accurately transfer AMs among the mobile nodes. The stationary node’s ID identifies each unique stationary node. The unit space ID, mapped to the stationary node’s ID, represents the geographical information of the area at which the stationary node is located. The ZigBee Personal Area Network (PAN) identifier, which changes according to the network formation, is used to deliver the AM to the sensor network layer, thereby making it necessary to maintain the middleware along with the stationary node’s ID. Finally, the mobile nodes’ IDs must be saved along with the corresponding stationary node’s information where the mobile nodes were detected in order to determine the most recent location of the mobile node.

As mentioned in the Introduction, the new architecture should support both legacy WSN services and the new mobile asset management service with minimum modifications. Thus, a stationary node requires the stationary-side middleware containing a manage mobility tier and service legacy senor network application. Regardless of new data flow for communication between mobile nodes, legacy WSN applications have to be serviced by the same data flow. As both data flows of the proposed architecture and legacy service share the same MAC layer for communication, the middleware should be located between the MAC layer and Network layer, thereby deciding where the received packet will be transferred to the Network layer or middleware. In conclusion, by inserting the middleware into the sensor nodes, we can reuse the legacy WSN network as a sensor network tier in the proposed architecture.

### 3.4. The Complete LAMD Sequence with Middleware Support

**Figure 7.**
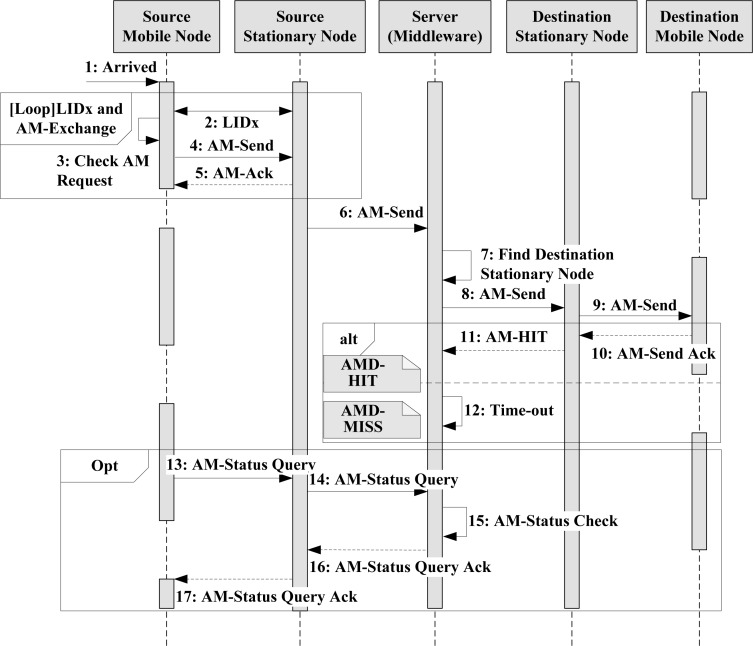
Complete LAMD sequence diagram

Transferring AMs are completed with middleware support which determines the target stationary node. Figure 7 describes the complete sequence used to send an AM. First, when mobile nodes are placed in a unit space (1), the stationary node initiates the LAMD protocol used to exchange its ID with the mobile nodes (2) and checks whether an AM is requested (3). If an AM is requested, the mobile node sends the AM-send packet (4). After acknowledging the mobile nodes (5), the stationary node sends the AM to the server through the sensor network (6). The server then initiates its decision making process for the target selection according to the middleware in order to determine the final destination mobile node for the AM (7). Once the target stationary is determined, the AM is directed to the unit space in which the stationary node is in charge (8); the stationary node is then responsible for sending the AM to the destination mobile node (9). Once the AM is successfully transferred to the final destination, the mobile node must to send an ack to inform the server of its arrival (10, 11). Otherwise, if the ack times out on the server (12), the server can perform an error-handling procedure. The handler may retry the above procedure or notify the source mobile node of the delivery failure. In some cases, the source mobile node may want to check whether the AM was successfully delivered. Although the middleware has responsibility for the missing AM and will thus retry or find another destination stationary node, sometimes the LAMD message might fail for several reasons. Therefore, if the AM contains urgent messages, the source mobile node needs to be notified of the result of the delivery so that it can take action, such as choosing another destination mobile node in accordance to the failed situation. This same source mobile node may send an AM-Status Query to the server (13, 14). The middleware that receives the request refers to the stationary node information table, which contains the result of the AM delivery. After learning the result (15), the middleware sends the result to the source stationary node (16). Finally, an AM-Status Query ack (17) can be sent to the source mobile node.

## 4. Experiment Description and Evaluation

### 4.1. Test Bed Organization

We developed a test bed consisting of 300 mobile nodes, four stationary nodes, four gateways, one server, and one smart tablet in order to evaluate the proposed mobile asset tracking system. The indoor service environment had an area of 50 m × 50 m, and the RF coverage of four stationary nodes was sufficient to cover the entire environment. The smart tablet was used to control the evaluation as well as identify the locations of the mobile nodes. The network stack employed Ethernet for the backbone network tier, ZigBee for the sensor network tier, and the LIDx and LAMD (IEEE 802.15.4 MAC) protocols for the mobility tier. The stationary nodes and mobile nodes used TI CC2430s, which employ an 8051 MCU core and a 2.4 GHz RF transceiver.

**Figure 8.**
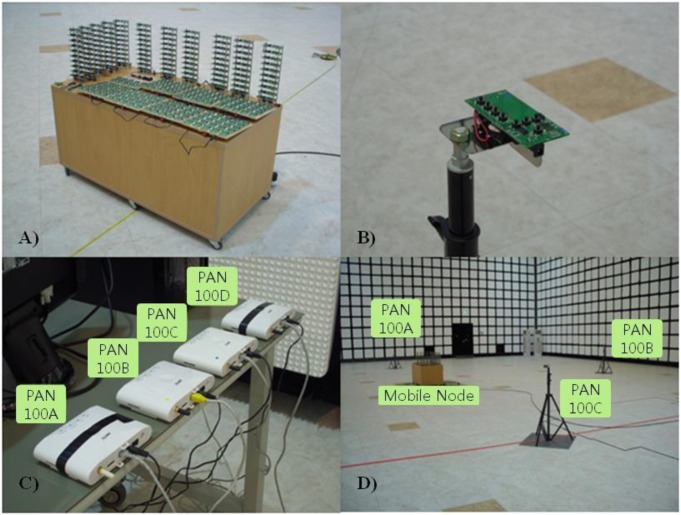
Test bed in the microwave anechoic chamber.

Figure 8 shows the implemented test bed in the mobile asset tracking system chamber. Figure 8(A) displays how the 300 mobile nodes were moved inside the environment. Figure 8(B) shows a stationary node installed in a fixed location on a tripod. Figure 8(C) shows the gateways connected to the server. Each gateway takes charge of a PAN in the sensor network tier. Figure 8(D) shows a view of the chamber. The configurations and results are discussed in Sections 4.2 to 4.5. Communication evaluation was processed both in the microwave anechoic chamber to prevent RF interferences and on a straight road near our office.

### 4.2. Communication Performance between Mobile and Stationary Nodes

**Figure 9.**
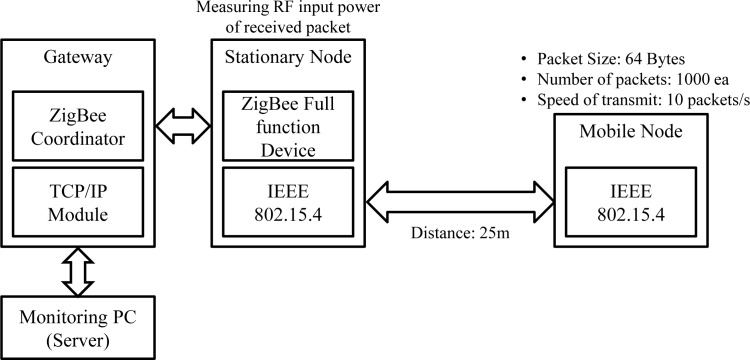
Experimental setup used to evaluate the successful packet transfer rate and RF input power.

We evaluated the successful packet transfer rate and RF input power according to both the changing dBm of the stationary nodes and the distance between the stationary and mobile nodes in the microwave anechoic chamber. Figure 9 describes the experimental setup. We set up a stationary node and a mobile node 25 m apart. The mobile nodes sent 64-byte packets per 100 ms to the gateway through the stationary node. Each packet included a sequence number. The gateway determined the RF input power, indicating receiver sensitivity, and sent it to the server. The server calculated the successful packet transfer rate by comparing the total expected number of packets (1,000) to the received number of packets. Seventeen output RF power levels were exploited.

Tables 1 and 2 summarize the results of the successful packet transfer rate and RF input power according to both the dBm of the stationary node and the distance between the stationary and mobile nodes, respectively. The results show that the minimum stationary node RF power needed for stable communication was −12 dBm; RF input power was −75.92. The stationary node was able to communicate at −14 dBm and −18 dBm, although the success rate decreased drastically. The maximum distance between the stationary and mobile nodes was 10 m for stable communication. When the distance was longer than 15 m, it became impossible for the stationary and mobile nodes to communicate at all.

### 4.3. Location-Awareness Evaluation

**Figure 10.**
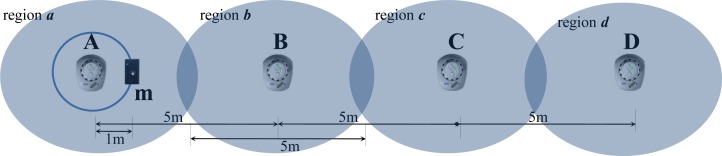
Setup used to measure location-awareness.

To evaluate the location-awareness of the mobile nodes, four stationary nodes were deployed equidistantly (5 m) and used for location reference. This evaluation was performed in the microwave anechoic chamber in order to prevent RF interference. Figure 10 shows the setup used to measure location-awareness. The LQI was measured in the mobile node located approximately 1 m from stationary node A. The LQI from A was the strongest; the signals showed relatively reduced strengths from positions B, C, and D according to the comparative physical distance from mobile node m. After analyzing the LQI values, we normalized them into five levels: original, neighbor 1 (NS 1), neighbor 2 (NS 2), neighbor 3 (NS3), and neighbor 4 (NS 4). The original showed the strongest LQI value and NS 4 had the weakest LQI value.

**Figure 11.**
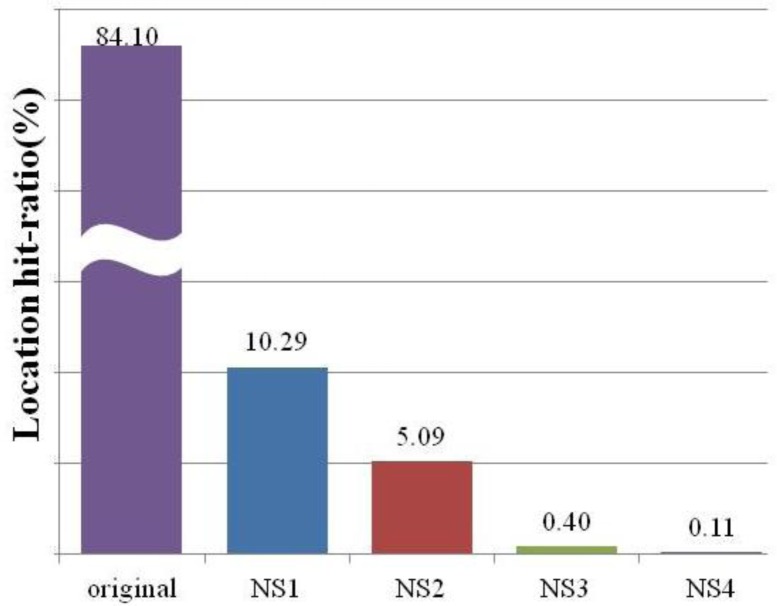
Average location hit-ratio using LIDx.

Figure 11 demonstrates the “I am in the Origin location. What is the measured location by LIDx and how accurate is it?” query. The results show that LIDx delivered an accurate location for the original 84.10% of the time, whereas NS 1 to 4 showed 10.29%, 5.09%, 0.40%, and 0.11% accuracies, respectively. Due to the reliability limitations of the link quality, the hit-ratio either decreased or increased. However, we believe that the average hit-ratio of the original + NS 1 approached 95%, which is acceptable for mobile asset tracking services.

### 4.4. System Stability under Numerous Mobile Nodes

To evaluate network stability while employing numerous mobile nodes in the proposed system, we conducted an experiment for 6 h in the microwave anechoic chamber using one stationary node and either 100 or 300 mobile nodes. Each mobile node sent 20-byte packets per 10 s to the stationary node; the packets included the sequence number. The server calculated the average successful packet transfer rate as well as the standard deviation by analyzing all packets received from the mobile nodes according to sequence number. The setup for this evaluation is similar to that shown in Figure 9.

Table 3 presents a summary of the network stability evaluation results. The results show that the average successful packet transfer rates were above 97.07% and 95.8% for 100 and 300 mobile nodes, w, whereas standard deviations were under 1.57 and 2.75, respectively, for the 300 mobile nodes. Even though the average successful packet transfer rate and standard deviation increased as time passed, we observed that the system was very stable over 6 h.

### 4.5. Successful Packet Transfer Rate according to Speed of Mobile Nodes

Mobile asset tracking systems must work stably and not fail due to fast moving mobile nodes operating at different speeds. In this experiment, we determined the successful packet transfer rate when mobile nodes moved at different speeds. For this experiment, we needed to secure current speed and keep constant velocity of mobile nodes. Therefore, we decided to use a car as vehicle of mobile nodes because it satisfied our requirements. Moreover, although our research was focused around indoor environments, this experiment reveals that proposed architecture is applicable outdoor as well as indoor.

**Figure 12.**
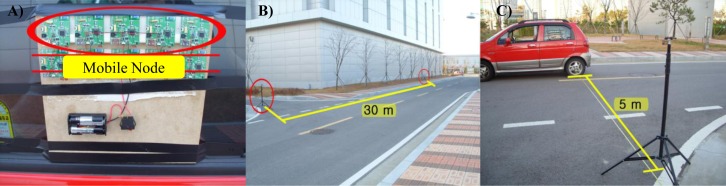
Setup used to evaluate the successful packet transfer rate according to changes in the mobile nodes’ speeds.

We attached five mobile nodes to a car and GPS used to verify the speeds of the mobile nodes. Four stationary nodes were installed at 30 m intervals on a roadside for communication. Similar to the above experiment, the server calculated the average successful packet transfer rate by analyzing all of the packets received from mobile nodes according to changes in the mobile nodes’ speeds. Figure 12 shows the setup used for this evaluation. Figure 12(A) shows the five mobile nodes attached to the car, Figure 12(B) shows the straight road where the evaluation was conducted. The distance between the car and the stationary node was 5 m, as shown in Figure 12(C).

**Figure 13.**
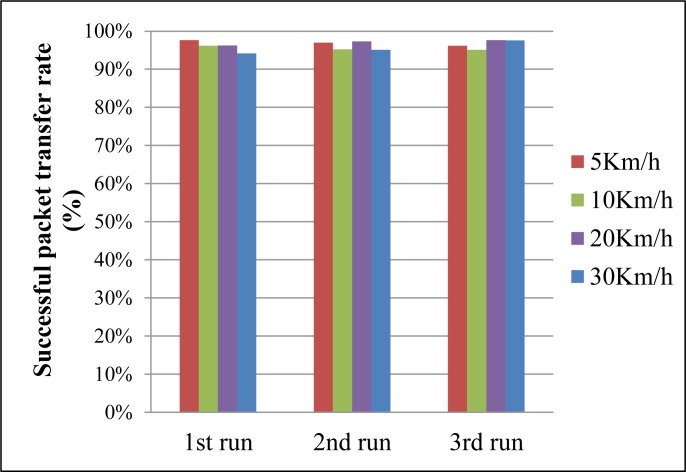
Average successful packet transfer rate according to changes in the mobile nodes’ speeds.

Figure 13 shows the results of the stability evaluation according to changes in the mobile nodes’ speeds. We varied the speed between 5 km/h and 30 km/h and performed three runs per trial. The results show that the average successful packet transfer rates were above 95% in all trials. The results indicate that the proposed system guarantees the mobility of mobile nodes between the average walking and running speeds of a person without performance degradation (average walking speed of a person: 5 km/h, average running speed of a person: 30 km/h).

## 5. Conclusions and Future Work

Here, we presented a mobile asset tracking system architecture and evaluated it thoroughly on a test bed. The proposed network architecture is composed of a mobility tier, a sensor network tier, and a backbone network tier according to their communication properties. For use as a communication protocol between the mobility and sensor network tiers, the LIDx and LAMD protocols were suggested for location-awareness of a massive number of mobile nodes and message deliveries regardless of location, which are hard to provide using legacy WSN techniques such as ZigBee. Middleware was developed for the server to transfer AMs accurately amongst mobile nodes and arbitrate between proposed service and legacy service. The evaluation was carried out in a microwave anechoic chamber and on a straight road near our office. We evaluated the performance of the communications between the mobile and stationary nodes, location-awareness performance, system stability under numerous mobile nodes, and system stability according to mobility of the mobile nodes. The accuracy of location-awareness was higher than 84%. The 300 mobile nodes showed a successful packet transfer rate higher than 95.8% and a standard deviation lower than 2.75%. Upon changing the mobile node speed, the architecture could support average successful packet transfer rates higher than 95% at 5 km/h, 10 km/h, 20 km/h, and 30 km/h. The results show that the proposed architecture is applicable to real-world mobile asset tracking services.

In future works, we will develop the middleware to decrease network congestion on the server paths and to support self-organizing services. The middleware also will provide novel features, such as resource discovery and service advertising in a distributed and self-organized manner. In the near future, various mobile asset management services based on WSN technology will play an important role in daily life. It is hoped that the proposed architecture will be used as a core technology for these future services.

## Figures and Tables

**Table 1.** Successful Packet Transfer Rate according to the Stationary Node dBm (distance: 25 m).

**dBm**	**The 1st try**		**The 2nd try**	
	
	**Successful transfer rate (%)**	**RF input power**	**Successful transfer rate (%)**	**RF input power**

4.5	99.7	−58.74	99.7	−58.28
2.5	99.7	−60.72	99.8	−63.04
1.0	99.8	−59.28	99.7	−60.72
−0.5	99.7	−62.86	99.7	−63.39
−1.5	99.8	−61.97	99.8	−62.92
−3.0	99.8	−65.16	99.7	−65.32
−4.0	99.7	−65.68	99.8	−69.30
−6.0	99.8	−69.21	99.7	−70.93
−8.0	99.7	−70.98	99.7	−71.98
−10	99.8	−74.41	99.7	−71.90
−12	99.7	−75.92	99.1	−76.12
−14	72.8	−79.11	69.4	−80.15
−16	58.9	−79.78	73.3	−80.13
−18	25.0	−80.34	11.4	−79.97
−20	0	-	0	-

**Table 2.** Successful Packet Transfer Rate according to Distance.

**Distance**	**The 1st try**		**The 2nd try**	
	
	**Successful Transfer Rate (%)**	**RF Input Power**	**Successful Transfer Rate (%)**	**RF Input Power**

2.5 m	99.7	−66.66	99.7	−64.94
5.0 m	99.8	−74.40	99.8	−76.44
7.5 m	99.7	−77.78	94.4	−79.11
10.0 m	99.8	−76.71	99.7	−75.99
12.5 m	71.6	−79.47	64.5	−79.85
15.0 m	0.0	-	0.0	-

**Table 3.** Average Successful Packet Transfer Rate according to Number of Mobile Nodes.

**Time**	**Successful packet transfer rate**			

	**100 Mobile Nodes**		**300 Mobile Nodes**	
	
	**Average (%)**	**Standard Deviation (%)**	**Average (%)**	**Standard Deviation (%)**

0 h 30 m	-	-	96.65	1.89
1 h 00 m	97.31	1.38	96.65	1.89
1 h 30 m	-	-	96.21	2.06
2 h 00 m	97.26	1.57	96.21	2.06
2 h 30 m	-	-	96.46	1.82
3 h 00 m	97.19	1.33	96.46	1.82
3 h 30 m	-	-	96.03	2.03
4 h 00 m	97.11	1.35	96.03	2.03
4 h 30 m	-	-	95.96	2.46
5 h 00 m	97.34	1.5	95.96	2.46
5 h 30 m	-	-	95.80	2.75
6 h 00 m	97.07	1.35	95.80	2.75
